# Dark exciton anti-funneling in atomically thin semiconductors

**DOI:** 10.1038/s41467-021-27425-y

**Published:** 2021-12-10

**Authors:** Roberto Rosati, Robert Schmidt, Samuel Brem, Raül Perea-Causín, Iris Niehues, Johannes Kern, Johann A. Preuß, Robert Schneider, Steffen Michaelis de Vasconcellos, Rudolf Bratschitsch, Ermin Malic

**Affiliations:** 1 Department of Physics, Philipps-Universität Marburg, 35032 Marburg, Germany; 2 Institute of Physics and Center for Nanotechnology, University of Münster, 48149 Münster, Germany; 3 Chalmers University of Technology, Department of Physics, 412 96 Gothenburg, Sweden

**Keywords:** Two-dimensional materials, Two-dimensional materials

## Abstract

Transport of charge carriers is at the heart of current nanoelectronics. In conventional materials, electronic transport can be controlled by applying electric fields. Atomically thin semiconductors, however, are governed by excitons, which are neutral electron-hole pairs and as such cannot be controlled by electrical fields. Recently, strain engineering has been introduced to manipulate exciton propagation. Strain-induced energy gradients give rise to exciton funneling up to a micrometer range. Here, we combine spatiotemporal photoluminescence measurements with microscopic theory to track the way of excitons in time, space and energy. We find that excitons surprisingly move away from high-strain regions. This anti-funneling behavior can be ascribed to dark excitons which possess an opposite strain-induced energy variation compared to bright excitons. Our findings open new possibilities to control transport in exciton-dominated materials. Overall, our work represents a major advance in understanding exciton transport that is crucial for technological applications of atomically thin materials.

## Abstract (web summary)

Strain engineering can manipulate the propagation of excitons in atomically thin transition metal dichalcogenides. Here, the authors observe an anti-funnelling behavior, i.e., the exciton photoluminescence moves away from high-strain regions, and attribute it to the dominating role of propagating dark excitons.

## Introduction

The mechanical exfoliation of atomically thin nanomaterials has initiated a new research field^1^. Since then, in particular transition metal dichalcogenides (TMDs) have been intensively studied^2–4^. The family of these truly two-dimensional materials exhibits a strong Coulomb interaction giving rise to the formation of excitons, which are Coulomb-bound electron-hole pairs. Having binding energies of a few hundreds of meV, they dominate the optoelectronic response of TMDs even at room temperature^3^. Recent studies have shown that besides the regular bright excitons accessible in optical spectra, dark excitonic states play a significant role for understanding the optical response and non-equilibrium dynamics in TMDs^5–8^. Here, the Coulomb-bound electrons and holes either exhibit an opposite spin or are located at different valleys (K, K${}^{\prime}$, Λ) in momentum space. The required spin-flip or momentum-transfer cannot be provided by photons, making these states dark and difficult to access in optical spectra. Very recently, momentum-dark KΛ excitons (with the hole localized at the K and the electron at the Λ valley) could be visualized in angle-resolved photoemission spectroscopy^9^.

Being atomically thin, flexible, and exhibiting strong absorption and ultrafast non-equilibrium dynamics, TMDs have been considered as promising candidates for next-generation opto-electronic applications including light-emitting, detecting, and harvesting devices^10^. Optical excitation, relaxation dynamics and transport of carriers represent the crucial many-particle mechanisms governing the efficiency of such devices. The optical response^8,11^ and non-equilibrium dynamics^12^ of excitons in TMDs have been well studied. Exciton transport, however, has still remained elusive. In conventional materials, external electrical fields are used to control the transport of optically excited carriers^13^. However, deeply-bound excitons as neutral particles (consisting of negatively charged electrons and positively charged holes) are only weekly affected by in-plane electrical fields, e.g., via Stark shifts and exciton dissociation. Only in the case of spatially separated interlayer excitons in van der Waals heterostructures, an electrical control of exciton transport has been demonstrated^14^ via the energetic tuning allowed by the dipole moment^15^. Thus, strain engineering has been introduced to manipulate the propagation of excitons in atomically thin semiconductors and could be exploited to e.g., boost the energy conversion efficiency in photovoltaics devices^16^. TMDs are remarkably sensitive to strain resulting in a large tunability of their energy landscape. In particular, strain was shown to significantly shift exciton resonances^17,18^. As a result, strain-induced energy gradients allow us to control and manipulate exciton propagation. Excitons have been observed to funnel up to a micrometer range towards spatial regions with the strongest strain gradient, where the energy is minimal^16,19–24^.

**Fig. 1:**
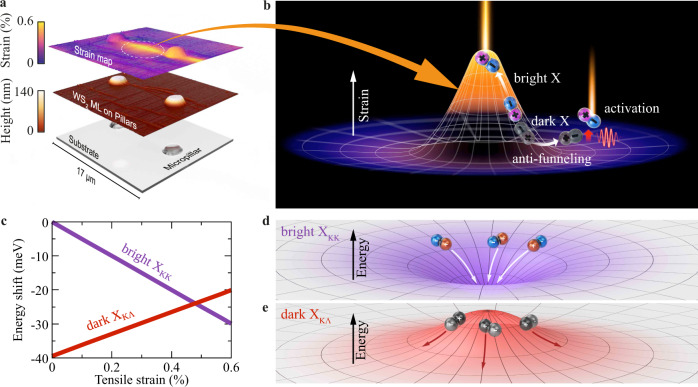
Dark and bright exciton funneling in an atomically thin semiconductor. **a** Creation of an inhomogeneous strain profile in a WS_2_ monolayer via transfer on an array of polymer micropillars. The lowest graph shows the patterened substrate, the middle one the sample topography with the WS_2_ monolayer, and the top one the resulting strain profile in the experiment. **b** Illustration of funneling of bright excitons and anti-funneling of dark excitons in the strained material. Dark excitons can be activated by phonons and/or defects making them visible in optical spectra. **c** Strain-induced shift of dark X_KΛ_ and bright X_KK_ exciton resonances. **d,e** Opposite shifts of *X*_KΛ_ and X_KK_ give rise to reverse spatial energy gradients resulting in an unconventional anti-funneling of dark X_KΛ_ excitons away from regions with maximum strain.

Tungsten-based TMD monolayers (WS_2_, WSe_2_) are indirect semiconductors, where dark excitons are the energetically lowest states^9,25^. Although these excitons are not directly accessible in optical spectra, they have been demonstrated to crucially influence optics, dynamics, and even diffusion in TMDs^3,4,26^. However, their role on exciton funneling has still remained in the dark. In this work, we combine spatially and temporally resolved photoluminescence (PL) measurements with a microscopic many-particle theory to investigate exciton funneling in presence of spatially inhomogeneous strain gradients in the exemplary case of a WS_2_ monolayer. Surprisingly, we find an inverse exciton funneling (*anti-funneling*) toward spatial regions with low strain (Fig. 1)—opposite to the conventional funneling behavior observed so far^20–24^. Shedding light on the many-particle processes behind the funneling process, we explain this counter-intuitive behavior. In presence of strain, lattice distortions induce valley-dependent variations of energies, i.e., bright and momentum-dark excitons shift in different directions (Fig. 1c). In particular, the energy of momentum-dark KΛ excitons increases with strain, and thus this type of excitons funnels opposite to the strain gradient toward spatial regions with minimal strain (Fig. 1e). Our joint experiment-theory study represents a major advance for the microscopic understanding of exciton funneling in atomically thin semiconductors including the entire landscape of bright and dark excitons.

## Results

### Spatiotemporal exciton funneling

**Fig. 2:**
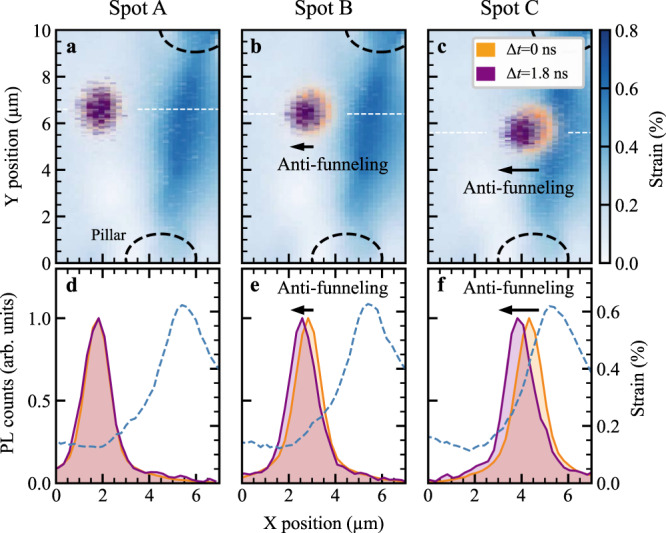
Exciton anti-funneling. **a**–**c** Spatially resolved photoluminescence from an inhomogeneously strained WS_2_ monolayer upon pulsed excitation at a delay time 0 ns (orange) and after 1.8 ns (purple). The blue colormap depicts the measured strain gradient induced by the micropillars. **d**–**f** Cuts along the *x* direction (horizontal white dashed line) in figures **a**–**c**. The dashed blue line shows the strain distribution along the *x* direction. We find that excitons do not propagate in absence of a strain gradient (spot A). Surprisingly, they funnel towards lower-strain regions (opposite to conventional funneling), when the excitation spot is subject to a considerably large strain gradient (spot B and C). The supplementary material includes PL movies further illustrating this surprising anti-funneling behavior.

We investigate spatiotemporal exciton dynamics in a strongly spatially dependent strain profile. To create such a strain gradient, we use the concept of a nanopatterned surface, which has previously been exploited to create single-photon emitters in TMD monolayers^27–29^. We transfer the WS_2_ monolayer on an array of polymer micropillars (120 nm height and 2 μm width) on a borosilicate substrate (cf. Methods and Supplementary Information). Mechanical strain strongly modifies the electronic band structure and shifts exciton resonances (Fig. 1b). As a result, the applied strain can be mapped by measuring the energetically lowest exciton energy in spatially resolved optical absorption spectra (cf. SI for more details). Stamping the monolayer onto the micropillar array creates an inhomogeneous strain profile with the highest strain values directly between two pillars (Fig. 2a), allowing us to study exciton propagation in spatial regions of the highest strain gradients without interfering with the micropillar.

To investigate the time-resolved exciton propagation in the experiment, we spatially map the photoluminescence (PL) from the WS_2_ monolayer with picosecond time resolution. We pump the monolayer with ultrashort laser pulses (≈200 fs) at 2.1 eV. An objective lens focuses the laser onto a fixed sample position. By scanning a second objective lens in transmission geometry, we image the PL distribution onto a streak camera. In that way, we achieve a temporal and a two-dimensional spatial resolution of the PL (cf. Methods for details on the experimental setup and measurement routine). From this data, we obtain ultrafast PL movies tracking exciton motion within the strain profile created by micropillars (see the movies in the SI). Figure 2a–c shows cuts of the spatiotemporal dynamics highlighting three different excitation spots (A, B, C). Here, the orange color illustrates the exciton PL created by optical excitation (0 ns), while purple corresponds to the drifted exciton PL due to the funneling after 1.8 ns (cf. Supplementary Video S1 for the whole evolution). The blue colormap represents the strain field induced by the micropillars. For better visualization of the funneling process, we also show the PL and the strain profile along the *x* axis, cf. Fig. 2d–f. Interestingly, due to the stamping procedure a maximum strain of 0.6% is reached at the center between two micropillars, rather than directly at the position of the pillars. In these regions, the monolayer is lying flat on the substrate, which ensures a homogeneous dielectric environment. First, we optically pump at a location where the strain profile is flat (spot A). As a result, we find no observable exciton propagation (orange and purple line overlap in Fig. 2d). At locations B and C, we excite excitons in spatial regions with a significant strain gradient (dashed line in Fig. 2d–f). Here, we find a clear exciton drift, which is larger for a higher strain gradient (cf. Fig. 2e, f, and Supplementary Information). Unexpectedly, we observe exciton funneling towards low strain regions in contrast to conventional funneling^20–24^.

### Dark-exciton anti-funneling in WS_2_

To be able to explain this intriguing and unexpected anti-funneling behavior, we complement our experiments with microscopic calculations of the spatiotemporal dynamics of excitons. Here, we exploit a generalized drift-diffusion equation for exciton densities *N*_*v*_(**r**, *t*) yielding

1 $${\dot{N}}_{v}({{{{{{{\bf{r}}}}}}}},t)= 	\;\nabla \left(\right.D({{{{{{{\bf{r}}}}}}}})\nabla {N}_{v}({{{{{{{\bf{r}}}}}}}},t)\left)\right.+\frac{1}{{k}_{B}T}\nabla \left(\right.D({{{{{{{\bf{r}}}}}}}}){N}_{v}({{{{{{{\bf{r}}}}}}}},t)\nabla {E}_{v}({{{{{{{\bf{r}}}}}}}})\left)\right.\\ 	-{\gamma }_{t}{N}_{v}({{{{{{{\bf{r}}}}}}}},t)+{\dot{N}}_{v}^{{{{{{{{\rm{th}}}}}}}}}({{{{{{{\bf{r}}}}}}}},t).$$

Here, we explicitly take into account the entire exciton landscape, where the valley index *v* includes the energetically lowest 1s states of bright and momentum-dark excitons (such as KΛ, KK’, ΓK). The total exciton density *N*(**r**, *t*) = ∑_*v*_ *N*_*v*_(**r**, *t*) is initially spatially localized with a Gaussian profile reflecting the confined optical excitation pulse in the experiment. The spatial dependence of all other quantities stems from the applied strain gradient *s*(**r**), which in particular affects exciton energies *E*_*v*_(**r**). The latter are obtained by solving the Wannier equation^7^ starting from the unstrained single-particle dispersion relation^5^ and adding strain-induced shifts^30^, cf. Methods and Supplementary Information. The spatial variation of energy (i) impacts the stationary diffusion coefficient *D*(**r**) by altering the occupation of single exciton states and opening/closing exciton-phonon scattering channels^26^ (see also Supplementary Information) and (ii) induces a drift force −∇*E*_*v*_(**r**) for excitons, see Methods. The first term in Eq. (1) corresponds to the standard Fick’s equation for diffusion, i.e., it leads to a spatial broadening of the exciton occupation *N*_*v*_ due to the optically induced spatial gradient (∇*N*_*v*_(**r**, *t*)). This occurs already for homogeneous (*s*(**r**) = *s*_0_) or vanishing strain (*s* = 0). The appearing diffusion coefficient *D*(**r**) is microscopically obtained via a full evolution of the Wigner function^26^, cf. the Supplementary Information. In the second term in Eq. (1), the energy *E*_*v*_ plays the role of a scalar potential, whose spatial variation induces a drift force driving excitons toward spatial regions with a minimal *E*_*v*_. This force is present only for spatially inhomogeneous strain and leads to exciton funneling (Fig. 1b). Since with larger strain *E*_*v*_ decreases for bright (*v* = KK) and increases for dark (*v* = KΛ) excitons (Fig. 1c), we expect an opposite funneling behavior (cf. Supplementary Information). As a result, momentum-dark KΛ excitons could provide an explanation for the anti-funneling behavior observed in the experiment (Fig. 2).

Previous studies have shown that the interaction with high-dipole molecules^31^ or phonons^8^ can brighten dark exciton states and make them visible and even dominate the low-temperature PL of tungsten-based TMDs. Through funneling, dark KΛ excitons can reach spatial regions, where the spectral separation of dark and bright states *E*_KK_ − *E*_KΛ_ becomes small, significantly enhancing the phonon-driven intervalley scattering. As a direct consequence, a large amount of originally dark KΛ excitons scatters into bright KK states and becomes visible in PL spectra (cf. Figs. 1b and SI). Additional defect-induced activation mechanisms could further boost the effect. More details on the theoretical approach (including the total decay rate *γ*_*t*_ and intervalley thermalization ${\dot{N}}_{v}^{{{{{{{{\rm{th}}}}}}}}}$ appearing in Eq. (1) as well as funneling-induced activation of dark excitons) can be found in the Supplementary Information.

**Fig. 3:**
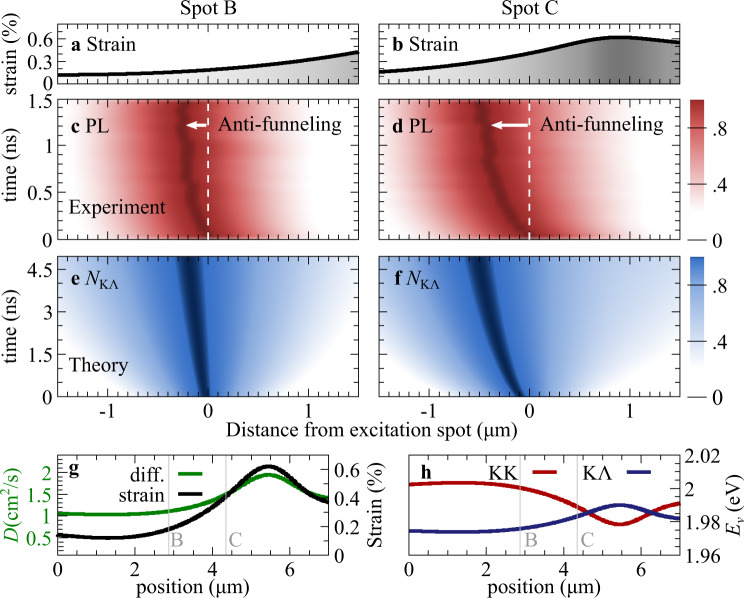
Exciton anti-funneling: experiment-theory comparison. **a,b** Spatial strain profiles at the spots B and C (illustrated in Fig. 2**b,c**). **c,d** Experiment: Spatiotemporal evolution of the PL signal imaged with the streak camera after an optical excitation at the spots B and C. **e,f** Theory: Temporally and spatially resolved dynamics of momentum-dark KΛ excitons obtained by solving the drift-diffusion dynamics [cf. Eq. (1)] assuming the same conditions as in the experiment. **g** Spatially dependent strain profile (black line, extracted from the experiment, cf. Fig. 2**e**) and theoretically predicted diffusion coefficient (green line) and **h** energy of bright KK and dark KΛ excitons. The position of the investigated spots B and C are denoted by thin vertical lines.

To further illustrate the anti-funneling behavior, we show the spatiotemporal evolution of the experimentally measured PL signal in Fig. 3c, d. We focus on spatial cuts along the *x* axis (cf. the dashed white line in Fig. 2b, c) for optical excitation close to the maximum strain gradient (corresponding to spot B and C). For a better orientation with respect to the funneling direction, we also plot the spatially dependent strain profile at these spots (cf. Fig. 3a, b). Opposite to the expected behavior, we see a clear leftward propagation away from regions with maximal strain, i.e., an *anti-funneling* of excitons. The propagation is more pronounced in the spot C due to the presence of a higher strain gradient. A quantitative analysis shows that excitons propagate for a few hundreds of nm within the first nanoseconds. This is sensitive to the strain profile, i.e., at spots with a larger strain gradient, the drift efficiency is higher (cf. the Supplementary Material). The anti-funneling length is in the same range as the conventional exciton propagation observed so far in literature^20,21^.

The PL at room temperature is usually associated with bright excitons^3,8^. However, these states cannot explain the observed anti-funneling behavior, since they propagate toward regions of high strain, where their energy is minimal (cf. Fig. 1c). In contrast, the energy of momentum-dark KΛ excitons decreases with strain and is thus the driving force for the observed anti-funneling behavior. To illustrate this, we show in Fig. 3e, f the spatially and temporally resolved occupation *N*_KΛ_ of momentum-dark KΛ excitons in direct comparison to the PL measurements in Fig. 3c, d. The theoretical data is obtained by numerically evaluating the drift-diffusion Eq. (1) exactly at the conditions of the experiment, i.e., assuming the same spatially inhomogeneous strain profile shown in Fig. 2e, f. In both experiment and theory, we find a clear exciton motion to the left of the excitation spot, i.e., towards spatial regions with a smaller strain gradient (cf. the strain profile in Fig. 3a, b). This indicates that the surprising anti-funneling observed in the PL can indeed be directly ascribed to the propagation of dark excitons. This can only occur at excitation spots, where momentum-dark KΛ excitons are energetically lower or close to the bright KK states (Fig. 3h) and thus exhibit a considerable occupation. We find a more pronounced funneling after optical excitation at location C closer to the maximum of the strain gradient, since here the spatial variation of strain is steeper, resulting in a stronger drift force −∇*E*_KΛ_(**r**). In addition, we observe both in experiment and theory that interestingly the funneling is initially faster and then it slows down approaching a stationary value for the propagation. This effect is well reproduced by the theoretically predicted evolution of dark-exciton occupation and can be understood as an interplay of two effects: First, the diffusion coefficient *D* is spatially-dependent and exhibits smaller values left from the excitation spots (cf. the green line in Fig. 3e). While funneling leftward, dark excitons move in regions with a smaller *D*, hence resulting in a decreasing funneling velocity. Second, dark excitons are subject to phonon-driven intervalley thermalization. A part of dark KΛ excitons scatters into KK states, which funnel to the right and effectively slow down the overall exciton funneling.

## Discussion

**Fig. 4:**
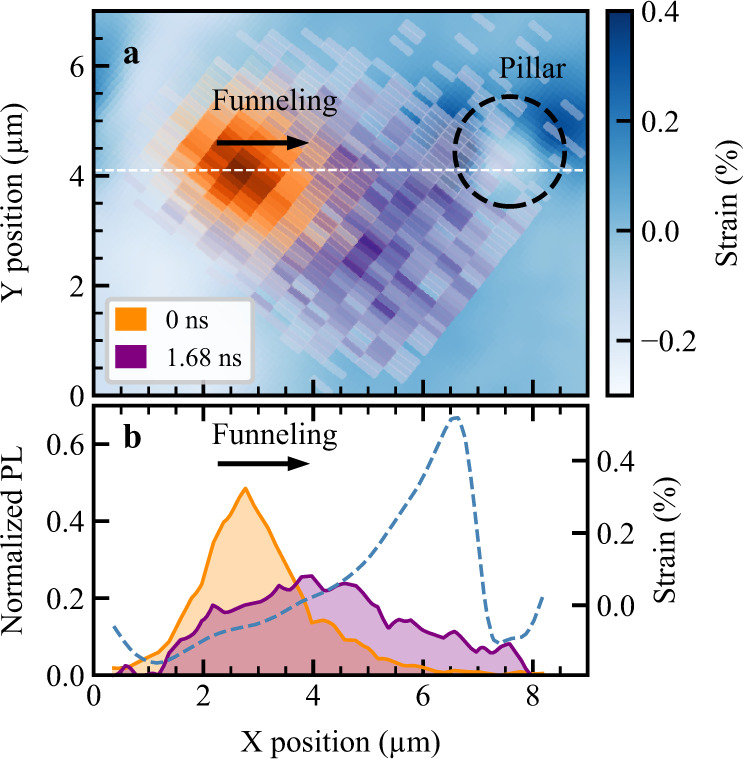
Conventional exciton funneling in MoSe_2_ monolayers. **a** Spatiotemporal photoluminescence of MoSe_2_ shown at Δ*t* = 0 ns (orange area) and for a delay time of 1.68 ns (purple area) in presence of a strain profile (blue scale). The dashed black circle denotes the position of the micropillar. **b** Cuts along the white dashed line in **a**. The dashed blue line shows the spatial strain profile. We find a conventional funneling towards region of higher strain in MoSe_2_ (see also Movie S2).

By combining spatiotemporal photoluminescence experiments with quantum-mechanic many-particle modeling we provide deep microscopic insights into the transport of excitons in atomically thin semiconductors. Creating a strain gradient via nanopatterned surfaces and investigating exciton propagation via spatiotemporal photoluminescence, we find an anti-funneling behavior in WS_2_ monolayers. Complementing our experiments with microscopic theory, we attribute the anti-funneling to dark KΛ excitons, which possess an opposite strain-induced energy variation compared to bright excitons and thus propagate in the opposite direction. To further prove that momentum-dark KΛ excitons are the origin of the inverse funneling observed in our experiments, we have performed similar measurements on a molybdenum diselenide (MoSe_2_) monolayer, cf. Fig. 4 and Supplementary Video S2. Contrary to the case of WS_2_, we find a conventional exciton funneling, i.e., the PL signal moves from low- toward high-strain regions (cf. orange and purple area indicating initial and final distributions, respectively). This observation is in full agreement with our theoretical predictions, since in MoSe_2_ dark KΛ excitons are located above the bright states^7^. Therefore, dark states are weakly occupied and are expected to have only a minor impact on exciton funneling (cf. Supplementary Information). Remarkably, a quantitative analysis of the funneling behavior shows an efficient propagation of excitons exceeding 1 μm within 0.8 ns (cf. Supplementary Information), which is beyond the reported values of some hundreds of nanometers in previous studies performed on WSe_2_^20,21^.

Our results demonstrate that strain engineering is an efficient tool to manipulate the propagation of neutral excitons. In particular, the interplay of dark and bright states offers new possibilities for tuning the optical response, dynamics, and transport, which are key processes for the realization of novel ultrathin and flexible optoelectronic devices.

## Methods

### Theoretical methods

The strain-dependent exciton energies entering Eq. (1) are obtained starting from the unstrained single-particle dispersion relation^5^, adding strain-induced shifts^30^ and solving on microscopic footing the Wannier equation^7,32^

2 $$\frac{{\hslash }^{2}{k}^{2}}{2{m}_{v}}{{{\Psi }}}_{v}({{{{{{{\bf{k}}}}}}}})-\mathop{\sum}\limits_{{{{{{{{\bf{q}}}}}}}}}{W}_{{{{{{{{\bf{q}}}}}}}}}{{{\Psi }}}_{v}({{{{{{{\bf{k}}}}}}}}+{{{{{{{\bf{q}}}}}}}})={E}_{v}^{{{{{{{{\rm{b}}}}}}}}}{{{\Psi }}}_{v}({{{{{{{\bf{k}}}}}}}}),$$

where *m*_*v*_ is the reduced exciton mass in exciton valley *v*. Here, ${E}_{v}^{{{{{{{{\rm{b}}}}}}}}}$ corresponds to the exciton binding energy, which depends weakly on strain via *m*_*v*_, cf. Table in Supplementary Information for the list of all appearing parameters. Furthermore, Ψ_*v*_(**k**) describes the excitonic wave function in momentum space, while *W*_**q**_ is the Coulomb interaction obtained via a modified form of the potential for charges in a thin film of thickness *d* surrounded by a dielectric environment^33^. Taking into account anisotropic dielectric tensors and solving the Poisson equation with the boundary conditions described above yields *W*_*q*_ = *V*_*q*_/*ϵ*_*scr*_(*q*) with the bare 2D-Fourier transformed Coulomb potential *V*_*q*_ and a non-local screening

3 $${\epsilon }_{scr}(q)={\kappa }_{1}\tanh \left[\frac{1}{2}\left[{\alpha }_{1}dq-{{{{{{\mathrm{ln}}}}}}}\,\left(\frac{{\kappa }_{1}-{\kappa }_{2}}{{\kappa }_{1}+{\kappa }_{2}}\right)\right]\right],$$

where ${\kappa }_{i}=\sqrt{{\epsilon }_{i}^{\parallel }{\epsilon }_{i}^{\perp }}$ and ${\alpha }_{i}=\sqrt{{\epsilon }_{i}^{\parallel }/{\epsilon }_{i}^{\perp }}$ account for the parallel and perpendicular component of the dielectric tensor *ϵ*_*i*_ of monolayer (i = 1)^34^ and environment (i = 2)^35^, see Table in Supplementary Information.

The exciton energy reads ${E}_{v}={E}_{v}^{0}-{E}_{v}^{{{{{{{{\rm{b}}}}}}}}}$, where ${E}_{v}^{0}$ is the minimal energy separation between conduction and valence band in valley *v*. This quantity depends crucially on strain^5,30^, see Table in Supplementary Information. Due to large spectral separations, we focus on the energetically lowest 1*s* states of the 6 most relevant exciton valleys (KK, KK${}^{\prime}$, KΛ, K${{{\Lambda }}}^{\prime}$, ΓK, ΓK${}^{\prime}$). These states exhibit a center-of-mass momentum **Q**, such that the state in valley *v* and momentum **Q** has an energy *E*_*v*,**Q**_ = *E*_*v*_ + *ℏ*^2^*Q*^2^/(2*M*_*v*_) with *M*_*v*_ being the total exciton mass. These energies depend strongly on strain mostly via *E*_*v*_, hence they are space-dependent in the case of a spatially-inhomogeneous strain profile, i.e., *E*_*v*,**Q**_ ≡ *E*_*v*,**Q**_(**r**). As explained in the main text, this induces (i) a spatially-dependent diffusion coefficient *D*(**r**) that has been microscopically evaluated in Ref. ^26^ (cf. Supplementary Information for more details) and (ii) a drift force −∇*E*_*v*_ with a generalization of the electrical mobility provided by *D*/*k*_*B*_*T* according to the Einstein relation^36,37^. It is well known that trions^38–41^ can also be observed in the presence of space-dependent strain profiles^22^, however, we focus here on the intrinsic undoped regime. The impact of spin-dark states^40,42^ has also been neglected, since they are expected to only lead to quantitative changes due to their similar character as KK${}^{\prime}$ excitons considered here.

In the presence of spatially-homogeneous strain, optically excited excitons reach an equilibrium characterized by a (local) thermalized energy distribution ${N}_{v}^{\circ }({{{{{{{\bf{r}}}}}}}},t)$^43^ and a conventional diffusive regime^44^. This occurs on a femtosecond timescale at room temperature. Since the timescale of exciton funneling is much longer, we have assumed that the stationary conditions are always fulfilled with ${N}_{v}^{\circ }({{{{{{{\bf{r}}}}}}}},t)$ corresponding to the Boltzmann distribution ${N}_{v}^{\circ }({{{{{{{\bf{r}}}}}}}},t)\propto {\sum }_{{{{{{{{\bf{Q}}}}}}}}}{g}_{v}{{{{{{{\rm{Exp}}}}}}}}\big[-{E}_{{{{{{{{\bf{Q}}}}}}}},v}({{{{{{{\bf{r}}}}}}}})/{k}_{B}T\big]$ with *g*_*v*_ as the valley-degeneracy factor (*g*_*v*_ = 3 for *v* = KΛ,K${{{\Lambda }}}^{\prime}$, otherwise *g*_*v*_ = 1). At every position, the relative amount ${\bar{n}}_{v}({{{{{{{\bf{r}}}}}}}})={N}_{v}^{\circ }({{{{{{{\bf{r}}}}}}}},t)/{\sum }_{v}{N}_{v}^{\circ }({{{{{{{\bf{r}}}}}}}},t)$ of exciton occupation in valley *v* with respect to the total exciton population ${\sum }_{v}{N}_{v}^{\circ }({{{{{{{\bf{r}}}}}}}},t)$ is time-independent, while it depends on space via the excitonic energies. In particular, the ratio ${\bar{n}}_{{{{{{{{\rm{K}}}}}}}}{{\Lambda }}}/{\bar{n}}_{{{{{{{{\rm{KK}}}}}}}}}$ of KΛ and KK excitons decreases for increasing strain due to the opposite strain-induced shifts of the exciton energy *E*_*v*_, cf. Fig. 1c in the main text.

We describe the spatiotemporal evolution of exciton densities *N*_*v*_(**r**, *t*) by exploiting the generalized drift-diffusion equation Eq. (1). As stated in the main manuscript, the first two terms provide diffusion and valley-intrinsic drift, respectively. The third term in Eq. (1) describes the decay of exciton occupation. Bright excitons localized in the light cone with a zero center-of-mass momentum decay via the radiative recombination rate *γ*_rad_ = 4.5 meV/*ℏ*, which was previously calculated^30^ and shows only small variations with strain. Once depleted via direct recombination, bright excitons are then refilled via scattering from states with a non-zero center-of-mass momentum. Since only a small amount of excitons resides in the light cone, the total excitonic density *N*(**r**, *t*) decays via an effective rate ${\gamma }_{{{{{{{{\rm{rad}}}}}}}}}{\bar{n}}_{{{{{{{{\rm{0}}}}}}}}}({{{{{{{\bf{r}}}}}}}})$ with ${\bar{n}}_{{{{{{{{\rm{0}}}}}}}}}$ being the fraction of the exciton occupation in the light cone at the position **r**. This results in a slower decay through radiative recombination of the overall excitonic density, which takes place on a timescale of tens of nanoseconds for tungsten-based monolayers at room temperature^43^. However, the PL signals decay typically much faster, suggesting that the leading decay mechanism in most TMDs is given by defects and impurities (except for hBN-encapsulated samples^45–47^). Since this is an extrinsic process and varies for each sample, we assume a typical value of *γ*_*t*_ = 1/*τ*, with *τ* = 0.35 ns in accordance with recent strain experiments on WS_2_^18^. The last term in Eq. (1) provides the intervalley thermalization which can be written as ${\dot{N}}_{v}^{{{{{{{{\rm{th}}}}}}}}}({{{{{{{\bf{r}}}}}}}},t)=\bar{\gamma }\left[{N}_{v}^{\circ }({{{{{{{\bf{r}}}}}}}},t)-{N}_{v}({{{{{{{\bf{r}}}}}}}},t)\right]$. The thermalization time $\bar{\tau }={\bar{\gamma }}^{-1}$ depends on exciton valley and strain (and as such being in principle space-dependent), however, it occurs on a femtosecond timescale at room temperature^43,44^. Compared to the much slower exciton diffusion and funneling taking place on a timescale of hundreds of ps to ns, we assume the thermalization to occur instantaneously at all spatial positions. Note that we have evaluated Eq. (1) along the *x* direction of strain variation exploiting the symmetry of the system.

### Sample and experimental setup

To create inhomogeneous strain fields in an atomically thin semiconductor, we transfer a TMD monolayer onto a borosilicate substrate (170  μm thickness) with an array of micropillars on top^28,29,48^. The arrays of polymer pillars (Novolak Allresist AR-N 7520.07, 10 μm distance between pillars) are fabricated using electron beam lithography. They have a height of 140 nm and circular or elliptical shapes with a diameter of ≈2 μm. To characterize the created strain landscape, we record spectrally resolved white-light transmission images of the sample and extract the shift of the bright X_KK_ exciton for every sample position (cf. Supplementary Information). The strongest exciton shifts appear between two pillars (*X*-direction). The exact strain profile depends on the varying experimental conditions (angle, pressure, shear forces) during the transfer of the TMD monolayer onto the pillars and, hence, varies from sample to sample. From these exciton shifts, we can extract the strain in the monolayer using the uniaxial gauge factor for monolayer WS_2_ (− 50 meV/%)^18^.

To measure the temporally and spatially resolved exciton motion, we use a home-built optical transmission microscope. Femtosecond pulses from a tunable Er:fiber laser (*E* = 2.1 eV, *τ* ≈ 200 fs, *H* = 0.9 μJ/cm^2^) or from a Ti:Sapphire laser (*E* = 1.71 eV, *τ* < 200 fs, *H* = 3.8 μJ/cm^2^) are focused onto the sample using an objective lens (Nikon TU Plan ELWD 50x, *NA* = 0.6) with a focus size <1 μm. The photoluminescence is collected by a second objective lens (Nikon TU Plan 100x, *NA* = 0.9) and imaged onto the entrance slit of a streak camera (Hamamatsu C10910-01) with an attached SCMOS sensor. Residual pump light is removed using an appropriate long pass filter. The streak camera provides temporal and spatial resolution along the X dimension. To obtain a spatial resolution in the Y dimension, the collection objective lens is mounted on a piezo positioner and moved in the *Y* direction. This configuration allows us to spatially map the photoluminescence by scanning the objective lens in the vertical direction, projecting different sample positions onto the entrance slit of the streak camera, while exciting the monolayer at a fixed position. In that way, we obtain ultrafast videos of the exciton motion in monolayer TMDs.

## Supplementary information

Supplementary Information

Description of Additional Supplementary Files

SupplementaryMovie1

SupplementaryMovie2

## Data Availability

The datasets generated during and/or analysed during the current study are available from the corresponding authors on reasonable request.

## Code availability

The codes used to generate the data are available from the corresponding authors on reasonable request.

## References

[1] Geim A, Grigorieva I (2013). Van der Waals heterostructures. Nature.

[2] Manzeli S, Ovchinnikov D, Pasquier D, Yazyev OV, Kis A (2017). 2D transition metal dichalcogenides. Nat. Rev. Mater..

[3] Wang G (2018). Colloquium: excitons in atomically thin transition metal dichalcogenides. Rev. Mod. Phys..

[4] Mueller T, Malic E (2018). Exciton physics and device application of two-dimensional transition metal dichalcogenide semiconductors. npj 2D Mater. Appl..

[5] Kormányos A (2015). k⋅p theory for two-dimensional transition metal dichalcogenide semiconductors. 2D Mater..

[6] Zhang X-X, You Y, Zhao SYF, Heinz TF (2015). Experimental evidence for dark excitons in monolayer WSe_2_. Phys. Rev. Lett..

[7] Selig M (2016). Excitonic linewidth and coherence lifetime in monolayer transition metal dichalcogenides. Nat. Commun..

[8] Brem S (2020). Phonon-assisted photoluminescence from indirect excitons in monolayers of transition-metal dichalcogenides. Nano Lett..

[9] Madéo J (2020). Directly visualizing the momentum-forbidden dark excitons and their dynamics in atomically thin semiconductors. Science.

[10] Mak KF, Shan J (2016). Photonics and optoelectronics of 2D semiconductor transition metal dichalcogenides. Nat. Photonics.

[11] Yu H, Cui X, Xu X, Yao W (2015). Valley excitons in two-dimensional semiconductors. Natl. Sci. Rev..

[12] Merkl P (2019). Ultrafast transition between exciton phases in van der Waals heterostructures. Nat. Mater..

[13] Xia F, Mueller T, Lin Y-m, Valdes-Garcia A, Avouris P (2009). Ultrafast graphene photodetector. Nat. Nanotechnol..

[14] Unuchek D (2018). Room-temperature electrical control of exciton flux in a van der Waals heterostructure. Nature.

[15] Ciarrocchi A (2019). Polarization switching and electrical control of interlayer excitons in two-dimensional van der Waals heterostructures. Nat. Photonics.

[16] Feng J, Qian X, Huang C-W, Li J (2012). Strain-engineered artificial atom as a broad-spectrum solar energy funnel. Nat. Photonics.

[17] Peng Z, Chen X, Fan Y, Srolovitz DJ, Lei D (2020). Strain engineering of 2D semiconductors and graphene: from strain fields to band-structure tuning and photonic applications. Light Sci. Appl..

[18] Niehues I (2018). Strain control of exciton–phonon coupling in atomically thin semiconductors. Nano Lett..

[19] Castellanos-Gomez A (2013). Local strain engineering in atomically thin MoS_2_. Nano Lett..

[20] Cordovilla Leon DF, Li Z, Jang SW, Cheng C-H, Deotare PB (2018). Exciton transport in strained monolayer WSe_2_. Appl. Phys. Lett..

[21] Moon H (2020). Dynamic exciton funneling by local strain control in a monolayer semiconductor. Nano Lett..

[22] Harats MG, Kirchhof JN, Qiao M, Greben K, Bolotin KI (2020). Dynamics and efficient conversion of excitons to trions in non-uniformly strained monolayer WS_2_. Nat. Photonics.

[23] Gelly, R. J. et al. Probing dark exciton navigation through a local strain landscape in a WSe_2_ monolayer. *arXiv preprint arXiv:2103.01064* (2021). 10.1038/s41467-021-27877-2 PMC8752834 35017506

[24] Koo, Y. et al. Tip-induced nano-engineering of strain, bandgap, and exciton funneling in 2D semiconductors. *Adv. Mater*., 2008234 (2021). 10.1002/adma.202008234 33709476

[25] Deilmann T, Thygesen KS (2019). Finite-momentum exciton landscape in mono- and bilayer transition metal dichalcogenides. 2D Mater..

[26] Rosati R (2021). Strain-dependent exciton diffusion in transition metal dichalcogenides. 2D Mater..

[27] Kern J (2016). Nanoscale positioning of single-photon emitters in atomically thin WSe_2_. Adv. Mater..

[28] Branny A, Kumar S, Proux R, Gerardot BD (2017). Deterministic strain-induced arrays of quantum emitters in a two-dimensional semiconductor. Nat. Commun..

[29] Palacios-Berraquero C (2017). Large-scale quantum-emitter arrays in atomically thin semiconductors. Nat. Commun..

[30] Khatibi Z (2018). Impact of strain on the excitonic linewidth in transition metal dichalcogenides. 2D Mater..

[31] Feierabend M, Berghäuser G, Knorr A, Malic E (2017). Proposal for dark exciton based chemical sensors. Nat. Commun..

[32] Brem S, Selig M, Berghaeuser G, Malic E (2018). Exciton relaxation cascade in two-dimensional transition metal dichalcogenides. Sci. Rep..

[33] Brem S (2019). Intrinsic lifetime of higher excitonic states in tungsten diselenide monolayers. Nanoscale.

[34] Laturia A, Van de Put ML, Vandenberghe WG (2018). Dielectric properties of hexagonal boron nitride and transition metal dichalcogenides: from monolayer to bulk. npj 2D Mater. Appl..

[35] Geick R, Perry CH, Rupprecht G (1966). Normal modes in hexagonal boron nitride. Phys. Rev..

[36] Hess O, Kuhn T (1996). Maxwell-Bloch equations for spatially inhomogeneous semiconductor lasers. I. Theoretical formulation. Phys. Rev. A.

[37] Jacoboni, C. *Theory of Electron Transport in Semiconductors: a Pathway from Elementary Physics to Nonequilibrium Green Functions*, vol. 165 (Springer Science & Business Media, 2010).

[38] Gao F (2016). Valley trion dynamics in monolayer MoSe_2_. Phys. Rev. B.

[39] Kato T, Kaneko T (2016). Transport dynamics of neutral excitons and trions in monolayer WS_2_. ACS Nano.

[40] Cadiz F (2018). Exciton diffusion in WSe_2_ monolayers embedded in a van der Waals heterostructure. Appl. Phys. Lett..

[41] Titze M, Li B, Zhang X, Ajayan PM, Li H (2018). Intrinsic coherence time of trions in monolayer MoSe_2_ measured via two-dimensional coherent spectroscopy. Phys. Rev. Mater..

[42] Molas MR (2017). Brightening of dark excitons in monolayers of semiconducting transition metal dichalcogenides. 2D Mater..

[43] Selig M (2018). Dark and bright exciton formation, thermalization, and photoluminescence in monolayer transition metal dichalcogenides. 2D Mater..

[44] Rosati R, Perea-Causín R, Brem S, Malic E (2020). Negative effective excitonic diffusion in monolayer transition metal dichalcogenides. Nanoscale.

[45] Raja A (2019). Dielectric disorder in two-dimensional materials. Nat. Nanotechnol..

[46] Cadiz F (2017). Excitonic linewidth approaching the homogeneous limit in MoS_2_-based van der Waals heterostructures. Phys. Rev. X.

[47] Ajayi OA (2017). Approaching the intrinsic photoluminescence linewidth in transition metal dichalcogenide monolayers. 2D Mater..

[48] Proscia NV (2018). Near-deterministic activation of room-temperature quantum emitters in hexagonal boron nitride. Optica.

