# Supplementary material for: Dark exciton anti-funneling in atomically thin semiconductors
Source: Nat Commun. 2021 Dec 10;12:7221. doi: 10.1038/s41467-021-27425-y (PMC8664915; doi:10.1038/s41467-021-27425-y)
Supplement: Supplementary file 1 — Supplementary Information [file 41467_2021_27425_MOESM1_ESM.pdf]

Supplementary Information for
Dark exciton anti-funneling in atomically thin semiconductors

Roberto Rosati,[1,*] Robert Schmidt,[2,*] Samuel Brem,[1] Raül Perea-Causín,[3] Iris Niehues,[2] Johannes Kern,[2] Johann A. Preuß,[2] Robert Schneider,[2] Steffen Michaelis de Vasconcellos,[2] Rudolf Bratschitsch,[2] and Ermin Malic[1,3]

[1]*Department of Physics, Philipps-Universität Marburg, 35032 Marburg, Germany*
[2]*Institute of Physics and Center for Nanotechnology, University of Münster, 48149 Münster, Germany*
[3]*Department of Physics, Chalmers University of Technology, Fysikgården 1, 41258 Gothenburg, Sweden*

1. STRAIN-DEPENDENT DIFFUSION COEFFICIENTS AND SIMULATION PARAMETERS

The strain-dependent energies entering Eq. (1) in the main manuscript are obtained by investigating homogeneous lattice deformations. In particular, the excitonic energies $E_v(\mathbf{r}) = E_v[s(\mathbf{r})]$ have been microscopically calculated by solving the Wannier equation [1]. Here, we started from the unstrained single-particle dispersion relation [2] and added strain-induced spectral shifts [3]. The used input parameters for our theoretical approach include single-particle energies and masses as well as dielectric constants and radiative and non-radiative decay rates, cf. Table I.

The spatial dependence of the diffusion coefficient, i.e. $D \equiv D(x)$, contributes to the predicted initially faster and subsequently slower propagation of excitons (cf. Fig. 3c-f of the main manuscript) and has been determined in a joint theory-experiment study of homogeneously strained TMD samples [4], cf. Fig. S1. The theoretical results (dark thick line in Fig. S1) have been obtained by calculating the spatiotemporal dynamics of excitons in Wigner representation reading

$$\dot{N}^v_{\mathbf{Q}}(\mathbf{r},t) = -\left(\frac{\hbar\mathbf{Q}}{M_v}\cdot\nabla + \gamma_{\text{rad}}\delta_{\mathbf{Q},0}\delta_{v,KK}\right)N^v_{\mathbf{Q}}(\mathbf{r},t) + \Gamma^{v;KK}_{\mathbf{Q};0}|p_0(\mathbf{r},t)|^2 + \dot{N}^v_{\mathbf{Q}}(\mathbf{r},t)\Big|_{th}. \tag{1}$$

Here, the Wigner distribution $N^v_{\mathbf{Q}}(\mathbf{r},t)$ summed over excitonic momenta $\mathbf{Q}$ provides the spatially dependent exciton density $N_v(\mathbf{r},t)$. It undergoes a free propagation (first term), which is counteracted by exciton-phonon coupling (last term). The latter is evaluated microscopically [5, 6], where the strength of the coupling is obtained from first-principle calculations [7]. The competition between these two terms results in an initial transient phase with time-varying *effective* diffusion coefficients $D \equiv D(t)$ that evolves into a stationary conventional diffusion $D(t) \equiv D$ [8]. The

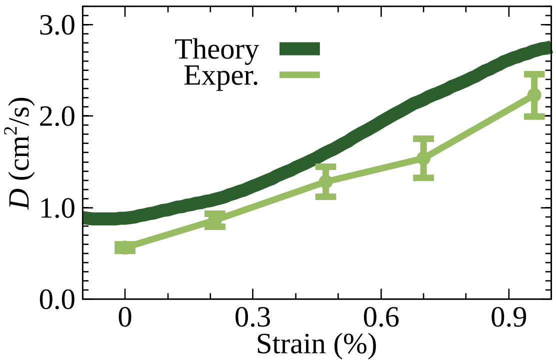

Fig. S 1: **Diffusion coefficients in homogeneously strained WS_2.** Diffusion coefficient plotted for a homogeneously strained WS_2 monolayer including a direct comparison between theoretically predicted (dark thick line) and experimentally measured (thin light line with error bars) values as a function of uniaxial strain [4].

---

*These authors contributed equally to this work.

duration of the transient phase crucially depends on temperature, ranging from tens of ps at very low temperatures down to hundreds of fs at room temperature [8]. The results shown in Fig. S1 are taken once the conventional diffusion is reached for different homogeneous strain values. Note that the second and the third term in Eq. (1) describe recombination of bright excitons and the phonon-assisted formation of incoherent excitons, respectively [4]. Although crucial for the optical response and formation dynamics of excitons, these processes either involve only a few states in the light-cone or occur on a much faster timescale and have thus a negligible impact on the stationary exciton diffusion.

While strain has only a minor effect on excitonic masses or radiative recombination [3, 9, 10], it strongly affects excitonic energies, whose variations can drastically change the efficiency of exciton-phonon scattering by opening/closing intervalley scattering channels. This results in an increase of the diffusion coefficient observed in Fig. S1, which is in excellent agreement with the values obtained experimentally upon bending of a WS_2 monolayer deposited on PMMA substrate (cf. the thin line with error bars in Fig. S1). Depending on strain, the measured values of the diffusion coefficient range from tens of cm^2/s up to a few cm^2/s - in agreement with previous experiments for tungsten-based TMDs on SiO_2 [11–13]. Note that SiO_2 has similar dielectric characteristics as the PMMA substrate employed in the experiment shown in Fig. S1 [4], thus allowing a direct quantitative comparison theory and experiment. The reported values are smaller than those obtained in the presence of hBN-encapsulation [14, 15]. This is expected due to the reduced dielectric disorder [16–18] and the increased contribution from electron-hole plasma playing a larger role due to the decreased exciton binding energy in hBN-encapsulated TMD samples [15].

Single-particle energies		
valley v	E_v^0 (eV)	$\partial_s E_v^0$ (meV/%)
KK	2.308	-52.8
KΛ	2.336	30.8
Single-particle masses		
valley, band	m (m_0)	$\partial_s m$ (m_0/%)
K, cond.	0.27	-0.006
Λ, cond.	0.64	0.0
K, val	-0.36	0.0045
Dielectric constants		
$\alpha_{WS_2} \approx 1.47$	$\kappa_{env.} \approx 9.26$	$\kappa_{env.} = 2.45$
Decay rates		
$\gamma_{rad} = 4.5\text{meV}/\hbar$	$\gamma_t \approx 2.9$ ns^{-1}	

Table I: **Input parameters for performed simulations.** Single-particle energies E_v^0, masses m and associated strain-induced variations $\partial_s E_v^0$, $\partial_s m$ [2, 3] induced by strain s for the valley v. Here, m_0 is the free-electron mass. Strain variations are adjusted by the factor stemming from the Poisson effect [4]. Further required parameters include dielectric constants of the TMD monolayer [19] and of the surrounding environment (taking an average of SiO_2 [20] and air) as well as radiative γ_{rad} [3] and total decay rates γ_t [21] (the latter dominated by non-radiative mechanisms).

2. LINEAR STRAIN PROFILE

The spatiotemporal dynamics of excitons under spatially-inhomogeneous strain profiles depends on strain both via its absolute value and its spatial gradient, cf. Eq. (1) of the main manuscript. The former affects the value of the diffusion coefficient D, see Sec. 1, or the relative population of a given exciton valley v, while the latter induces the force $-\nabla E_v(x)$. Here, we assume a linear strain profile $s = \bar{s} + x\partial_x s$ with both $\bar{s}$ and $\partial_x s$ being space independent. In this way the derivative $\partial_x s$ of the strain s is constant at all spatial points. Hence, the driving force $-\nabla E_v(x)$ does not depend on the position, remaining in particular identical while excitons move in different regions. Note that linear strain profiles can typically be found further away from the maximum strain gradient produced by a pillar. Here, we choose the values of $\bar{s}$=0.5% and $\partial_x s_i$=0.5%/μm, cf. the black line in Fig. S2(a).

We start with an initial total exciton occupation $N(x, 0)$ corresponding to a Gaussian centered at $x_0 = 0$ with a FWHM of 1μm. The strain profile gives rise to a spatial variation of the diffusion coefficient $D(x)$ (green line in Fig. S2(a)) and to spatial shifts of excitonic energy E_v (Fig. S2(b)). The latter are obtained via the Wannier equation, while D depends on position via strain $s(x)$, i.e. $D(x) \equiv D(s(x))$, cf. Sec. 1. We find that E_{KK} decreases with strain, while $E_{K\Lambda}$ shows the opposite behaviour. This results in different drift forces -$\partial_x E_v$ of approximately 280 eV/cm for KK and -180 eV/cm for KΛ excitons. In Fig. S2(c), we show the PL profile $I(x, t) \propto \gamma_{rad}\bar{n}_0(x)N(x, t)$, which depends on the total exciton population $N(x, t)$ weighted by the radiative decay rate γ_{rad} and the fraction of

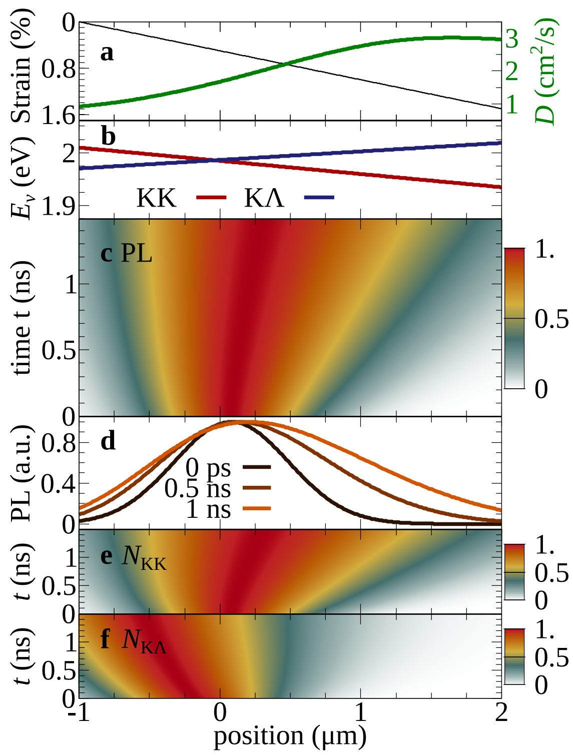

Fig. S2: **Exciton funneling under linear strain.** (a) Linear strain profile (black line) and resulting diffusion coefficient D (green line). (b) Spatial change of the energy minima of KK and KΛ excitons. (c) Spatiotemporal evolution of the normalized photoluminescence (PL) after an initial Gaussian excitonic distribution centered at $x = 0\mu$m with a FWHM of 1μm. (d) Snapshots of the PL at fixed times. (e-f) Spatiotemporal dynamics of bright KK and momentum-dark KΛ excitons, respectively.

the exciton occupation $\bar{n}_0(x)$ populating the light cone at the position x. At t=0, the spatial PL profile is centered slightly on the right-hand side of $x = x_0 \equiv 0$, where the initial excitonic distribution (as generated by a laser pulse) is centered, cf. also the PL time cuts in Fig.S2(d). This can be traced back to a higher exciton occupation in the light cone $\bar{n}_0(x)$ for $x > x_0$ due to the lower energetic position of KK vs. KΛ excitons in this region (cf. Fig. S2(b)).

The time evolution of the PL illustrates the expected exciton funneling toward spatial regions with higher strain values following the drift force $-\partial_x E_{\mathrm{KK}}$ for the bright KK excitons. During the funneling, the PL spatial profiles become asymmetric, displaying a more pronounced right flank, cf. Fig. S2(d). This stems from the interplay of the spatial variation of the diffusion coefficient D and the occupation of bright excitons $\bar{n}_0$. The overall behaviour of the PL follows the evolution of the distribution of bright KK excitons, cf. Fig. S2(e). In contrast, momentum-dark KΛ excitons show a qualitatively different behaviour demonstrating an anti-funneling toward regions of smaller strain values (Fig. S2(e)) - in clear contrast to N_{KK} excitons and the behaviour observed in the PL. This reflects the opposite spectral shift in presence of strain and thus opposite drift forces $-\partial_x E_{\mathrm{KK}}$ and $-\partial_x E_{\mathrm{K\Lambda}}$ for KK and $K\Lambda$ excitons. However, we do not see any signatures of anti-funneling in the PL, since no activation mechanism for dark excitons have been considered here.

3. ACTIVATION OF DARK EXCITONS

We now study a scenario, where momentum-dark KΛ excitons funneling away from regions of maximum strain can be visualized in spatiotemporal PL. To this end, we consider a Gaussian strain profile $s(x) = s_0 + s_i \exp\left(-x^2/(2\sigma^2)\right)$ with $s_0 = 0.6\%$, $s_i = -1.2\%$ and a FWHM of 4μm (black line in Fig. S3a). Such a strain profile induces a repulsive potential for KK and an attractive one for KΛ excitons, cf. Fig. S3b. The choice of the strain profile induces a peculiar behaviour of the diffusion coefficient D, which shows minima at approximately $x = \pm 1\mu$m corresponding to a strain of 0%. We start with an initial optically-excited exciton density that is spatially centered at $x_0 = 1\mu$m close to the maximum of the strain gradient. The initial stage of the PL evolution is as expected: The PL moves toward the regions of maximal strain magnitude s $(x > x_0)$ reflecting the funneling of KK excitons, cf. Fig. S3c. However,

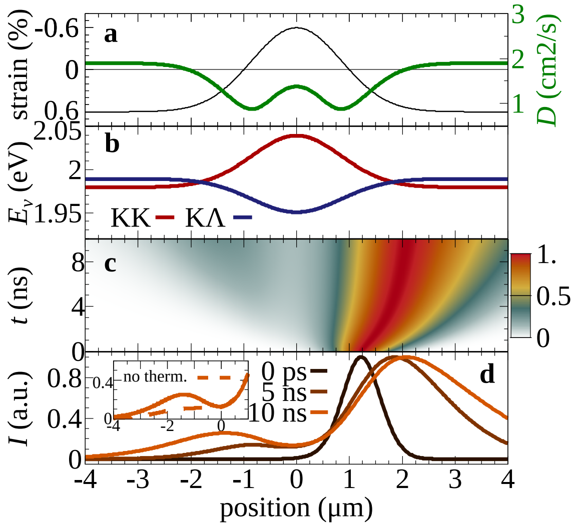

Fig. S3: **Activation of dark excitons through funneling.** The same as in Fig. S2, but now using a Gaussian strain profile. We find a time-delayed formation of a new peak for negative x values that can be ascribed to momentum-dark excitons. The inset demonstrates the crucial role of intervalley exciton thermalization for the activation of dark states.

at later times a new peak appears on the left side $x < x_0$, as demonstrated in Fig. S3d showing time-cuts of the spatiotemporal PL. The origin of this peak can be traced back to the presence of KΛ excitons, whose distribution $N_{K\Lambda}$ moves towards the region of smallest strain at $x = 0$. Then, a fraction of KΛ excitons keeps on diffusing further to the left (due to the first term in Eq. (1) of the main manuscript). When they reach the spatial region $x \lesssim -1\mu$m, where $E_{KK} < E_{K\Lambda}$ (Fig. S3b), KΛ excitons scatter into energetically lower KK states, from where they can emit light. Thus, the interplay of exciton funneling and intervalley thermalization activates originally dark excitons and makes them visible in spatiotemporal PL profiles.

The crucial role of the intervalley thermalization for the activation of dark excitons is demonstrated in the inset of Fig. S3d, where we show the PL profile without intervalley scattering (dashed line). The additional peak at negative x vanishes, since the amount of bright excitons funneling in this spatial region from the initial excitation at $x_0 = 1\mu$m is negligibly small. Given the large spatial extent of the investigated strain profile, dark excitons have to funnel a relatively long distance (from $+1$ to -1 μm) before being activated. Thus, the time delay for the formation of the second peak is relatively long with a few nanoseconds. The delay time can be in principle reduced by considering spatially narrower strain gradients. Furthermore, additional activation mechanisms for momentum-dark KΛ excitons beyond the considered phonon-driven intervalley scattering could further boost the effect. Here, in particular defect-induced activation could play an important role and needs to be further investigated [22, 23].

4. MEASURED EXCITON PROPAGATION: EXCITON FUNNELING VS ANTI-FUNNELING

To obtain a better understanding of the observed anti-funneling behaviour, in Fig. S4 we quantitatively analyze the propagation length and its direction with respect to the maximum of the strain profile in WS_2 and $MoSe_2$ monolayers. In the case of the conventional funneling towards spatial regions with maximal strain, we define the propagation as positive.

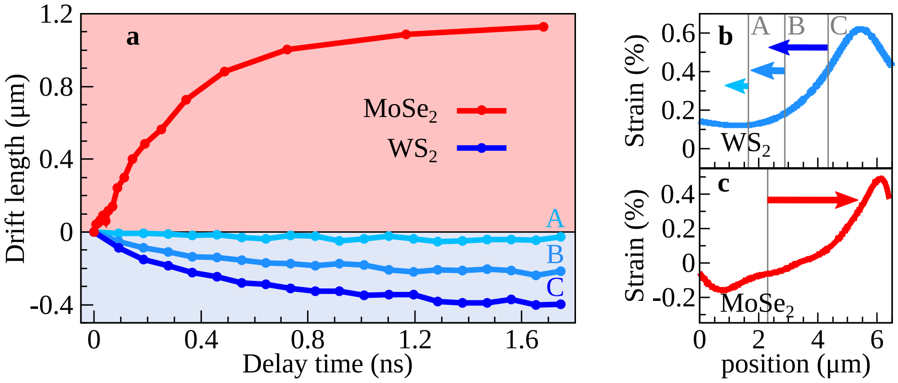

Fig. S4: **Strain-induced exciton propagation in $MoSe_2$ and WS_2 monolayers.** (a) Evolution of the central position of the PL intensity in $MoSe_2$ and WS_2 monolayers. The propagation length is evaluated at three different excitation spots located at different positions of the strain profile as illustrated in (b) for WS_2 and (c) $MoSe_2$. Regular exciton funneling results in a positive propagation in $MoSe_2$, i.e. excitons propagate towards regions of high strain (cf. the red arrow in (c)). Excitons propagate over a distance of 1 μm after 0.8 ns. In contrast, the anti-funneling behaviour induces a negative drift in WS_2 (cf. the blue arrows in (b)). The latter considerably increases when moving from spot A to C reflecting the enhanced strain gradient.

To quantify the exciton propagation, we evaluate the temporal evolution of the central position of the PL as extracted using a Gaussian fit of the experimental results. For the case of $MoSe_2$, we find a positive propagation, i.e. directed towards maximum strain (cf. Figs. S4a,c) - in agreement with with previous experiments [24, 25]. We observe a very efficient exciton propagation exceeding 1 μm already within the first nanosecond. In the case of WS_2, we first observe the crucial change of the sign in the direction of the propagation (cf. the blue lines in Fig. S4a), indicating an anti-funneling behaviour toward spatial region of minimal strain (cf. Fig. S4b). The propagation length is in the range of a few hundreds of nm within the first ns. This is less efficient compared to the regular funneling in

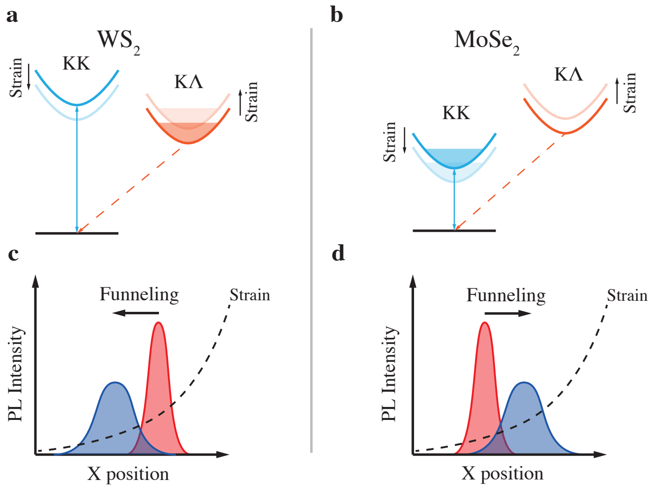

Fig. S5: **Schematics illustration of opposite funneling direction in WS_2 and $MoSe_2$.** Schematic energy alignment and strain dependence of KK and KΛ excitons in (a) WS_2 and (b) $MoSe_2$ monolayers. Energetically lower excitons carry majority of the population. (c)- (d) Opposite exciton funneling in the two monolayer materials due to the dominating KΛ (KK) excitons in WS_2 ($MoSe_2$) monolayers.

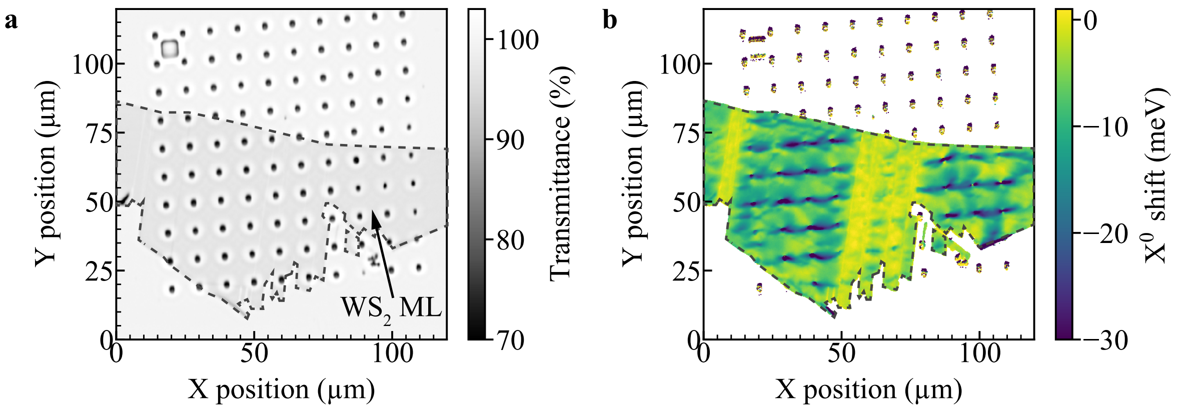

Fig. S6: **Sample structure.** (a) Optical transmittance micrograph of the WS_2 monolayer, transferred onto the micropillar array. (b) Map of the energy shift of the bright X_{KK} exciton under strain. The strongest red shift is observed between the pillars (x direction).

$MoSe_2$. However, we find a pronounced increase of the exciton propagation, when moving the excitation from spot A to C, i.e. closer to the maximum of the strain profile. Here, the strain gradient is higher resulting in a larger exciton drift, cf. Fig. S4b.

The exciton funneling direction in $MoSe_2$ is opposite to WS_2, which can be explained by the energetic alignment of bright KK and momentum-dark KΛ excitons, see Figs. S5a-d. In $MoSe_2$, KΛ excitons lies energetically much higher than the bright KK states. As a result, their occupation is small and their impact on the diffusion behaviour in $MoSe_2$ negligible. Here, bright excitons play the dominant role, where the reduction in energy with strain creates a spatial energy landscape favoring exciton propagation in direction of spatial regions with maximal strain, cf. also Fig. 1d in the main manuscript. The situation is drastically different in WS_2, where the momentum-dark KΛ excitons are the energetically lower states carrying the majority of the occupation. As a result, dark excitons govern the diffusion behavior. Here, the energy is enhanced with increasing strain creating a drift force in the opposite direction toward spatial regions with low strain, cf. also Fig. 1e in the main manuscript.

5. COMPARISON OF SAMPLE TOPOGRAPHY AND STRAIN

The strain is characterized by recording spectrally resolved optical transmission images of the sample (Fig. S6a) and extracting the shift of the bright X_{KK} exciton for every sample position in Fig. S6b. In Fig. S7, we compare the 3D profile of the WS_2 monolayer stamped onto the micropillars with the resulting strain profile. Figure S7a depicts an atomic force microscopy (AFM) image of the relevant sample area. The height image shows that the monolayer conforms to the micropillars and the flat substrate in-between, except for a few nanoscopic folds. The strain (Fig. 7b) has its maximum in the middle between two pillars, which is due to the experimental conditions during the transfer of the monolayer. This sample allows us to measure the exciton motion in a strain gradient, where the monolayer is in contact with the substrate. Therefore, we can rule out that other effects, such as changes of the dielectric environment have a significant influence on the spatial change of exciton energies.

Finally, we illustrate the strain profiles created for WS_2 and $MoSe_2$ monolayers, cf. Fig. 8. The crosses indicate the excitation spots. The strain profile in WS_2 is essentially straight and orthogonal to the (leftward) anti-funneling direction of excitons. In the case of $MoSe_2$, the strain profile has a more complicated shape exhibiting a maximum close to the pillar followed by a drawn-out tail.

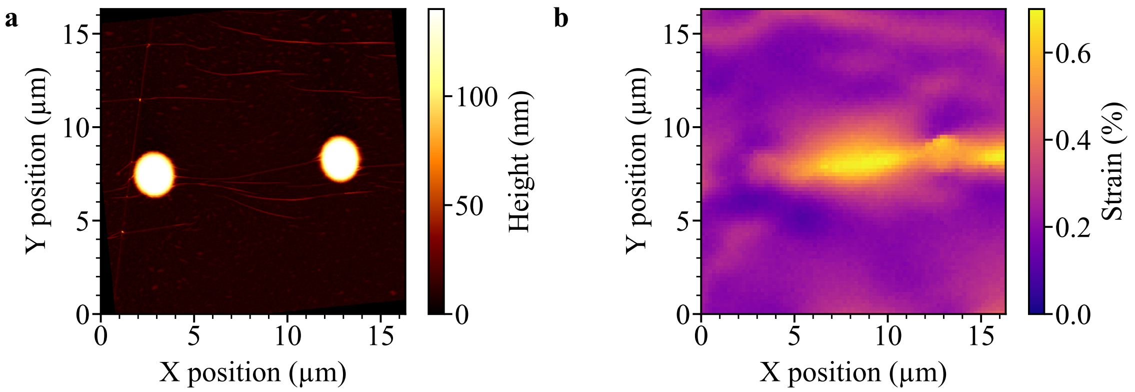

Fig. S7: **Comparison of sample topography and strain.** (a) Atomic force micrograph (AFM) of the WS_2 monolayer on the micropillar substrate. The monolayer conforms to the pillars and lies flat on the substrate in-between the pillars. Only small nanoscopic folds appear in the area between the pillars due the non-uniform strain. (b) Measured strain map of the same sample area. Due to the stamping conditions, the strongest tensile strain appears in the region between the two pillars.

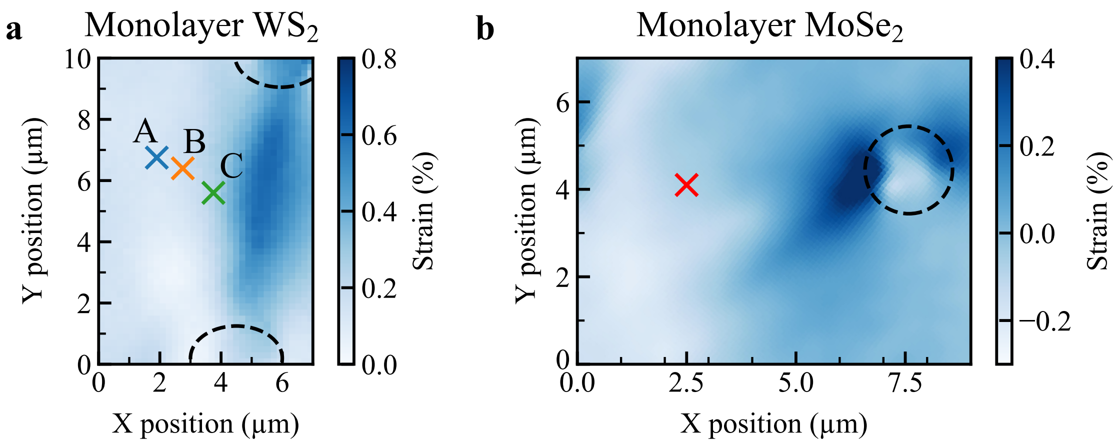

Fig. S8: **Strain profiles.** Strain profiles in (a) WS_2 and (b) $MoSe_2$ monolayers with crosses indicating the different excitation spots investigated in the main part, cf. Fig. 2 and 4 in the main manuscript.

[1] M. Selig, G. Berghäuser, A. Raja, P. Nagler, C. Schüller, T. F. Heinz, T. Korn, A. Chernikov, E. Malic, and A. Knorr, "Excitonic linewidth and coherence lifetime in monolayer transition metal dichalcogenides," *Nat. Commun.* **7**, 13279 (2016).

[2] A. Kormányos, G. Burkard, M. Gmitra, J. Fabian, V. Zólyomi, N. D. Drummond, and V. Fal'ko, "k·p theory for two-dimensional transition metal dichalcogenide semiconductors," *2D Mater.* **2**, 022001 (2015).

[3] Z. Khatibi, M. Feierabend, M. Selig, S. Brem, C. Linderälv, P. Erhart, and E. Malic, "Impact of strain on the excitonic linewidth in transition metal dichalcogenides," *2D Mater.* **6**, 015015 (2018).

[4] R. Rosati, S. Brem, R. Perea-Causín, R. Schmidt, I. Niehues, S. M. de Vasconcellos, R. Bratschitsch, and E. Malic, "Strain-dependent exciton diffusion in transition metal dichalcogenides," *2D Mater.* **8**, 015030 (2021).

[5] M. Selig, G. Berghäuser, M. Richter, R. Bratschitsch, A. Knorr, and E. Malic, "Dark and bright exciton formation, thermalization, and photoluminescence in monolayer transition metal dichalcogenides," *2D Mater.* **5**, 035017 (2018).

[6] S. Brem, M. Selig, G. Berghaeuser, and E. Malic, "Exciton relaxation cascade in two-dimensional transition metal dichalcogenides," *Sci. Rep.*, **8**, 8238 (2018).

[7] Z. Jin, X. Li, J. T. Mullen, and K. W. Kim, "Intrinsic transport properties of electrons and holes in monolayer transition-metal dichalcogenides," *Phys. Rev. B* **90**, 045422 (2014).

[8] R. Rosati, R. Perea-Causín, S. Brem, and E. Malic, "Negative effective excitonic diffusion in monolayer transition metal

dichalcogenides," *Nanoscale* **12**, 356 (2020).

[9] O. B. Aslan, M. Deng, and T. F. Heinz, "Strain tuning of excitons in monolayer WSe_2," *Phys. Rev. B* **98**, 115308 (2018).

[10] M. Feierabend, A. Morlet, G. Berghäuser, and E. Malic, "Impact of strain on the optical fingerprint of monolayer transition-metal dichalcogenides," *Phys. Rev. B* **96**, 045425 (2017)

[11] M. Kulig, J. Zipfel, Nagler, S. Blanter, C. Schüller, T. Korn, N. Paradiso, M. M. Glazov, and A. Chernikov, "Exciton diffusion and halo effects in monolayer semiconductors," *Phys. Rev. Lett.* **120**, 207401 (2018).

[12] S. Mouri, Y. Miyauchi, M. Toh, W. Zhao, G. Eda, and K. Matsuda, "Nonlinear photoluminescence in atomically thin layered wse_2 arising from diffusion-assisted exciton-exciton annihilation," *Phys. Rev. B*, **90**, 155449 (2014).

[13] L. Yuan, T. Wang, T. Zhu, M. Zhou, and L. Huang, "Exciton dynamics, transport, and annihilation in atomically thin two-dimensional semiconductors," *J. Phys. Chem. Lett.* **8**, 3371 (2017).

[14] F. Cadiz, C. Robert, E. Courtade, M. Manca, L. Martinelli, T. Taniguchi, K. Watanabe, T. Amand, A. C. H. Rowe, D. Paget, B. Urbaszek, and X. Marie, "Exciton diffusion in WSe_2 monolayers embedded in a van der Waals heterostructure," *Appl. Phys. Lett.* **112**, 152106 (2018).

[15] J. Zipfel, M. Kulig, R. Perea-Causín, S. Brem, J. D. Ziegler, R. Rosati, T. Taniguchi, K. Watanabe, M. M. Glazov, E. Malic, and A. Chernikov, "Exciton diffusion in monolayer semiconductors with suppressed disorder," *Phys. Rev. B* **101**, 115430 (2020).

[16] A. Raja, L. Waldecker, J. Zipfel, Y. Cho, S. Brem, J. D. Ziegler, M. Kulig, T. Taniguchi, K. Watanabe, E. Malic, T. F. Heinz, T. C. Berkelbach, and A. Chernikov, "Dielectric disorder in two-dimensional materials," *Nat. Nanotechnol.* **14**, 832 (2019).

[17] F. Cadiz, E. Courtade, C. Robert, G. Wang, Y. Shen, H. Cai, T. Taniguchi, K. Watanabe, H. Carrere, D. Lagarde, M. Manca, T. Amand, Renucci, S. Tongay, X. Marie, and B. Urbaszek, "Excitonic linewidth approaching the homogeneous limit in MoS_2-based van der Waals heterostructures," *Phys. Rev. X* **7**, 021026 (2017).

[18] O. A. Ajayi, J. V. Ardelean, G. D. Shepard, J. Wang, A. Antony, T. Taniguchi, K. Watanabe, T. F. Heinz, S. Strauf, X.-Y. Zhu, and J. C. Hone, "Approaching the intrinsic photoluminescence linewidth in transition metal dichalcogenide monolayers," *2D Mater.* **4**, 031011 (2017).

[19] A. Laturia, M. L. Van de Put, and W. G. Vandenberghe, "Dielectric properties of hexagonal boron nitride and transition metal dichalcogenides: from monolayer to bulk," *npj 2D Mater. Appl.* **2**, 6 (2018).

[20] R. Geick, C. H. Perry, and G. Rupprecht, "Normal modes in hexagonal boron nitride," *Phys. Rev.* **146**, 543 (1966).

[21] I. Niehues, R. Schmidt, M. Drüppel, P. Marauhn, D. Christiansen, M. Selig, G. Berghäuser, D. Wigger, R. Schneider, L. Braasch, R. Koch, A. Castellanos-Gomez, T. Kuhn, A. Knorr, E. Malic, M. Rohlfing, S. Michaelis de Vasconcellos, and R. Bratschitsch, "Strain control of exciton–phonon coupling in atomically thin semiconductors," *Nano Lett.* **18**, 1751 (2018).

[22] Y. Ou, Z. Kang, Q. Liao, S. Gao, Z. Zhang, and Y. Zhang, "Point defect induced intervalley scattering for the enhancement of interlayer electron transport in bilayer MoS_2 homojunctions," *Nanoscale* **12**, 9859 (2020).

[23] K. Kaasbjerg, K. S. Thygesen, and K. W. Jacobsen, "Phonon-limited mobility in n-type single-layer MoS_2 from first principles," *Phys. Rev. B* **85**, 115317 (2012).

[24] D. F. Cordovilla Leon, Z. Li, S. W. Jang, C.-H. Cheng, and B. Deotare, "Exciton transport in strained monolayer WSe_2," *Appl. Phys. Lett.* **113**, 252101 (2018).

[25] H. Moon, G. Grosso, C. Chakraborty, C. Peng, T. Taniguchi, K. Watanabe, and D. Englund, "Dynamic exciton funneling by local strain control in a monolayer semiconductor," *Nano Lett.* **20**, 6791 (2020).
