# Supplementary material for: Dark exciton anti-funneling in atomically thin semiconductors
Source: Nat Commun. 2021 Dec 10;12:7221. doi: 10.1038/s41467-021-27425-y (PMC8664915; doi:10.1038/s41467-021-27425-y)
Supplement: Supplementary file 2 — Description of Additional Supplementary Files [file 41467_2021_27425_MOESM2_ESM.pdf]

Title: SupplementaryMovie1

Description: Movie illustrating exciton antifunneling in WS_2 (cf. Fig. 2 in the main manuscript).

Title: SupplementaryMovie2

Description: Movie illustrating exciton antifunneling in $MoSe_2$ (cf. Fig. 4 in the main manuscript).
